# Endovascular Repair of Abdominal Aortic and Iliac Aneurysms Complicated by Multiple Endoleaks, Renal Dysfunction, and Urological Interventions

**DOI:** 10.7759/cureus.93743

**Published:** 2025-10-02

**Authors:** Julia Tarnowska, Kamil Stepkowski, Oskar Gąsiorowski, Jerzy Leszczynski, Zbigniew Gałązka

**Affiliations:** 1 Department of General, Vascular, Endocrine and Transplant Surgery, Medical University of Warsaw, Warsaw, POL; 2 Department of Pediatric Surgery, Medical University of Warsaw, Warsaw, POL

**Keywords:** abdominal aortic aneurysm, acute kidney injury, endoleak, endovascular procedures, nephrostomy, percutaneous, postoperative complications, ureteral obstruction, ureteral stents, urologic diseases

## Abstract

Abdominal aortic aneurysm (AAA) is a serious vascular condition often treated with endovascular aneurysm repair (EVAR). While EVAR is less invasive than open surgery, it may lead to complications, particularly in patients with risk factors. Acute kidney injury (AKI), endoleaks, and ureteral obstruction are among the most severe. The uniqueness of this case lies in the rare coexistence of multiple severe complications - recurrent endoleaks, renal dysfunction, and irreversible urological damage, occurring together in a single patient.

We present a case of a 61-year-old man with chronic kidney disease stage 4, atherosclerosis, atrial fibrillation, hypertension, and prior myocardial infarctions, who presented with abdominal aortic and iliac artery aneurysms. Initial EVAR with a bifurcated stent graft was successful. Three months later, complications began, including recurrent AKI, type IB and later type IA endoleak, type II endoleak, renal artery stent occlusion, hemorrhagic complications, and obstructive uropathy. Management included multiple reinterventions: nephrostomy placement, selective embolization, and relining of the renal artery stent via axillary access. Despite episodes of anuria and the need for dialysis, renal perfusion was partially restored following final stenting. Follow-up scintigraphy confirmed improved renal function.

This case demonstrates the complexity of managing EVAR complications in patients with advanced renal dysfunction and systemic vascular disease. It underscores the importance of multidisciplinary care, early recognition of endoleaks, and proactive renal protection strategies to improve patient outcomes.

## Introduction

Abdominal aortic aneurysm (AAA) is defined as a progressive, focal dilation of the abdominal aorta, characterized by an increase in vessel diameter by at least 150% compared to the normal diameter of the anatomically comparable portion of the artery [1-3]. According to published ultrasound data, the normal diameter of the infrarenal abdominal aorta typically measures up to 21 mm in men and 19 mm in women, with lower boundaries reported as low as 9 mm and 8 mm, respectively [4].

The strongest risk factor for AAA development is smoking. Other well-established risk factors include atherosclerosis, coronary artery disease, male sex, advanced age (>65 years), hypertension, dyslipidemia, ethnicity, and family history of AAAs [1, 2].

Endovascular aneurysm repair (EVAR) is the first-line treatment for AAA in many patients due to its minimally invasive nature and reduced preoperative morbidity compared to open repair. However, inadequate management - whether due to delayed diagnosis, suboptimal intervention, or preoperative - can lead to serious adverse outcomes. Short-term complications may include acute kidney injury (AKI), colonic ischemia, wound infections, myocardial infarction, and pneumonia. Long-term risks involve endoleaks, graft infections, aortoenteric fistulas, buttock claudication, and sexual dysfunction. For this reason, lifelong surveillance with imaging is essential to monitor for delayed complications and graft integrity [2].

Effective management necessitates a multidisciplinary approach, careful assessment of the risks and benefits of each treatment strategy, and long-term follow-up to ensure early identification and management of complications. This case highlights the importance of comprehensive care and long-term monitoring in patients with complex AAA presentations.

## Case presentation

Patient history and background

A 61-year-old male patient was referred to the Department of General, Vascular, Endocrine, and Transplant Surgery of the Medical University of Warsaw for management of abdominal aortic and iliac artery aneurysms. His medical background included active smoking, hypertension, hyperlipidemia, atherosclerosis of the aorta, paroxysmal atrial fibrillation, chronic kidney disease (CKD), gout, and a family history of coronary artery disease. He had also experienced two previous myocardial infarctions.

Six years earlier, during an abdominal ultrasound performed due to elevated renal function parameters, bilateral aneurysms of the abdominal aorta and iliac arteries were first identified. Laboratory investigations revealed elevated creatinine and urea levels and markedly decreased eGFR. A full summary of laboratory abnormalities and their progression over time is presented in Table 1.

**Table 1:** : Summary of Key Laboratory Investigations During the Clinical Course ↑ - Increased; ↓ - Decreased; – - Not measured or not available. eGFR: estimated glomerular filtration rate; EVAR: endovascular aneurysm repair

Parameter	Reference Range	Before First EVAR	Post-EVAR Peak AKI (27 Months after the Initial EVAR)	Post-Stabilization (31 months after the Initial EVAR)	Follow-up (Scintigraphy) (32.5 Months after the Initial EVAR)
Creatinine	0.6–1.3 mg/dL	↑ 5.94	↑ 10.8	↓ 2.1	–
Urea	15–50 mg/dL	↑ 198	↑ 245	–	–
eGFR	>60 mL/min/1.73 m²	↓ 9.9	↓ 5	↑ 31	↑ 28

Acute kidney injury was diagnosed, secondary to obstructive uropathy.

Initial imaging and endovascular treatment

Ultrasound revealed a patent abdominal aorta measuring 45 mm below the superior mesenteric artery, aneurysmally dilated to 56 mm, with mural thrombi. The iliac arteries were also dilated (left: 36 mm; right: 23 mm). No signs of rupture were present.

CT angiography confirmed aneurysmal dilation and detected an occlusion in the left iliac artery. An endovascular stent graft (bifurcated, nitinol, with suprarenal fixation, Jotec E-tegra) was successfully implanted via bilateral groin access at the referring institution. The procedure was uncomplicated, resulting in the complete exclusion of aneurysmal flow. Postoperative recovery was uneventful, aside from a red blood cell transfusion due to anemia. AKI was managed with hemodialysis and left nephrostomy, which was maintained for six months.

Disease progression and first complications

Three months later, follow-up Doppler ultrasound revealed an increase in aneurysm size (aortic: 62 × 55 mm; right iliac: 42 mm; left iliac: 39 mm). The patient reported right-sided lumbar pain, chills, and weakness, which later resolved spontaneously. Subsequent ultrasound showed left kidney cysts and right-sided renal pelvic dilatation (17 mm).

Four months later, angio-CT revealed a type IB endoleak from the right external or internal iliac artery (REIA/IIA) with retrograde flow to the aneurysm sac. Aneurysms of the right (53 mm) and left (45 mm) internal iliac arteries were noted. The inferior mesenteric artery (IMA) was occluded, with collateral perfusion via the arc of Riolan.

Progressive aneurysmal disease and type iliac artery (IA) Endoleak

Eighteen months post-initial surgery, abdominal MRI showed a saccular aneurysm of the aorta extending to the common iliac arteries. Measurements at bifurcation reached 60 × 68 mm, with left and right iliac aneurysms measuring 44 × 44 mm and 60 × 70 mm, respectively. A type IA endoleak was suspected, with aneurysm sac filling visible on the right side (Video 1).

**Video 1:** In CT, contrast leak was visible into the aneurysm sac along the entire length of the stent graft from the level of the aortic bifurcation and along the right stent graft limb, consistent with a type Ib endoleak. The beginning of the leak visibility is marked with a purple arrow.

An additional stent graft was implanted into the abdominal aorta (Jotec) with uneventful recovery.

Renal complications and urological interventions

One month later, renal function deteriorated severely. Imaging revealed right hydronephrosis due to suspected stent graft compression of the right ureter and ischemia of the left kidney. As the patient presented with advanced renal failure requiring urgent decompression, percutaneous nephrostomy was selected, since it provides faster and more reliable drainage than retrograde ureteral stenting in such acute settings.

A right nephrostomy was complicated by active hemorrhage from the renal puncture site and bladder, requiring embolization of the right renal artery and transfusion. Three units of red blood cells were administered due to anemia caused by this active bleeding. Despite hemodynamic stabilization, renal parameters remained profoundly deranged (Table 1). Inflammatory markers were increased (CRP 41 mg%, procalcitonin 3.7 ng/mL), and antibiotic therapy was initiated.

A left renal artery stent was implanted via axillary access, temporarily restoring urine output, but it soon occluded. Hemodialysis was resumed. A double-J stent was placed in the right ureter, though nephrostomy had to be reopened due to recurrent obstruction.

Scintigraphy showed an eGFR of 21 mL/min/1.73 m², with 65% contribution from the left kidney and 35% from the right.

Further vascular interventions and outcomes

**Figure 1:**
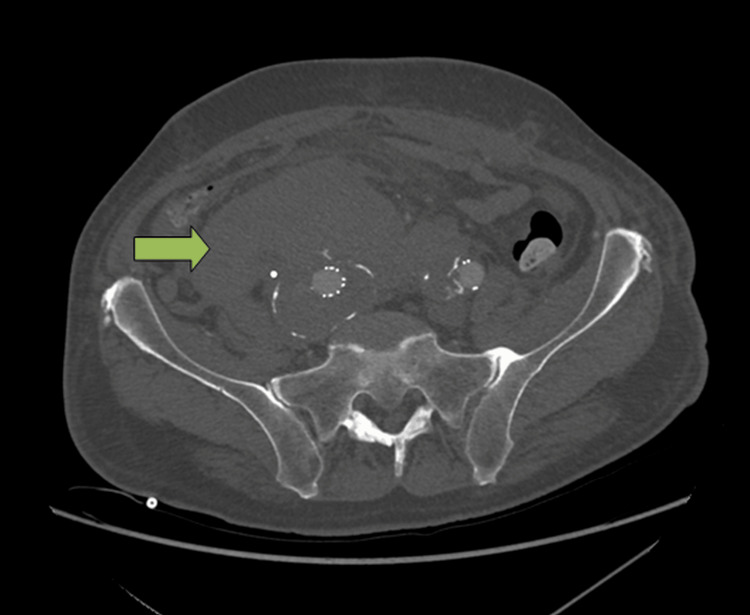
CT showed a large right-sided retroperitoneal hematoma (green arrow) extending from the level of the duodenum to the pelvis. The hematoma was adjacent to the aneurysm sac.

Four months later, admission to the Department of General, Vascular, Endocrine, and Transplant Surgery of the Medical University of Warsaw was due to an aneurysm of the abdominal aorta and iliac arteries. Overall, three vascular procedures were performed: Surgery 1: Laparotomy, aneurysmotomy, thrombus removal, partial excision of aneurysm sac, ligation of the right internal iliac artery, and aneurysmorrhaphy around the stent graft; Surgery 2 (5 days later): Left renal artery stent revision with Astron Pulsar 5×60 mm stent and balloon post-dilatation; Surgery 3 (5 days later): CT revealed a right retroperitoneal hematoma with active bleeding near the DJ stent (Figure 1).

Emergency VBX XL 8 × 79 mm stent implantation into the right limb of the stent graft extending into the right external iliac artery was performed, along with laparotomy and hematoma evacuation.

Upon discharge, the patient had normal diuresis with improved renal parameters, including decreased serum creatinine and increased eGFR, relative to previous values (Table 1).

1.5 months later, the JJ catheter was removed, and the right nephrostomy was left in place. Scintigraphy showed an eGFR of 28 mL/min/1.73 m² (left kidney 81%, right 19%). Due to irreversible ureteral damage, reconstruction was not feasible. The right kidney showed only minimal perfusion and marginal parenchymal function. Under these circumstances, ureteral reconstruction would not have provided any meaningful clinical benefit, and permanent nephrostomy was maintained as the only option.

Recurrence of symptoms and further surgical management

**Figure 2:**
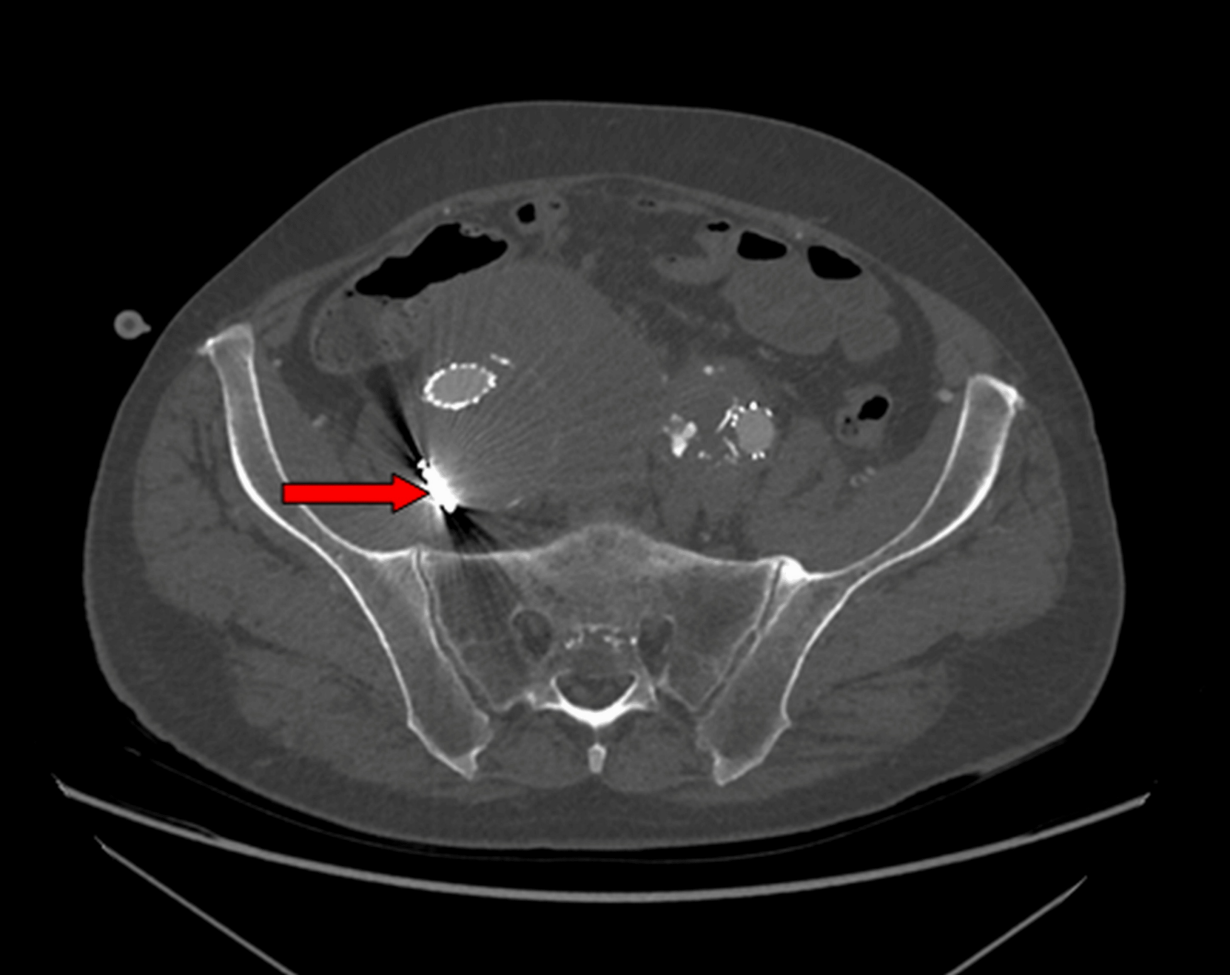
CT showed status post endovascular recanalization of renal artery stents and embolization of the right internal iliac artery (red arrow).

Two years later, the patient was readmitted with lower abdominal pain, low-grade fever, and a history of urinary retention. Angio-CT showed an enlarging aneurysm, type II endoleak from the right internal iliac artery, and a retroperitoneal collection. Embolization and percutaneous drainage were performed. During hospitalization, progressive deterioration of renal function was observed. Follow-up angio-CT revealed occlusion of previously implanted stents in the left renal artery. Endovascular stent recanalization was successfully carried out, with an uneventful periprocedural course (Figure 2).

**Figure 3:**
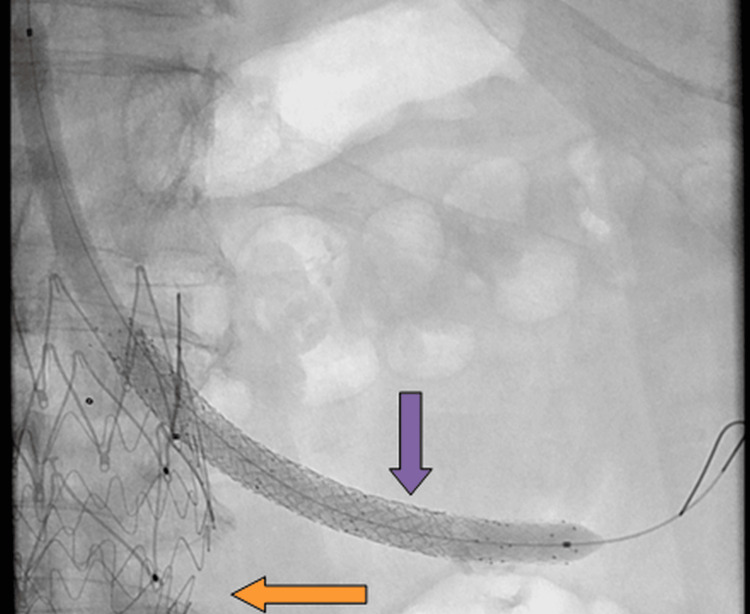
Angiography demonstrated recanalization of the left chimney to the left renal artery using a steerable sheath. Additional relining was performed with a Pulsar T3 6×80 mm stent, followed by lumen optimization with a 6 mm balloon (purple arrow). Reperfusion of the left renal parenchyma was achieved. A total of three stents (orange arrow) and a balloon (purple arrow) were visible. The stent graft began in the thoracic aorta, connected to the previous abdominal graft, and included branches to the visceral arteries.

One week after that, the patient exhibited recurrence of lower abdominal pain, low-grade fever, and urinary retention. Angio-CT revealed occlusion in the left renal artery. Endovascular recanalization with a stent Pulsar T3 6 × 80 mm (Figure 3), restoring urine output.

His condition worsened, and angio-CT suggested rupture of an AAA. Emergency endovascular repair with a branched thoracoabdominal stent graft was performed for a juxtarenal rupture due to a type IA endoleak (Video 2).

**Video 2:** : Status post-implantation of a branched stent graft with relining of the previous EVAR system and stent grafting of visceral and renal branches. Final angiography showed a probable type IV endoleak between the new and old systems, with no other endoleaks present. EVAR: endovascular aneurysm repair

The patient was stabilized. In the following days, inflammatory markers increased, and peritoneal signs appeared in the lower abdomen. A decision was made to perform a laparotomy and retroperitoneal hematoma drainage. A small, well-localized perforation of the sigmoid colon was identified without signs of fecal contamination or diffuse peritonitis. The perforation was probably caused by local ischemia and pressure necrosis from the adjacent hematoma. Therefore, primary suture repair was performed, and a colostomy was not considered necessary. The abdominal cavity was drained. Follow-up CT revealed appropriate coverage.

Parenteral nutrition was initiated. Inflammatory parameters normalized, diuresis was approximately 2-3 liters per day, and oral nutrition was initiated. The patient was discharged 20 days after the laparotomy, in good general condition and without symptoms (Video 3).

**Video 3:** Axial contrast-enhanced follow-up CT image of the abdomen demonstrating a thoracoabdominal stent graft with branches to the visceral arteries. The stent graft is correctly positioned with a patent lumen. Branches to the celiac trunk, superior mesenteric artery, and renal arteries are patent. No evidence of endoleak or stent graft migration.

## Discussion

EVAR is a commonly used method for treating AAAs. It is less invasive compared to classical surgical repair [2]. Although associated with lower early postoperative morbidity and mortality, EVAR is not free from complications. Among the most significant are AKI and endoleaks, both of which occurred in this patient and ultimately required multiple reinterventions [5, 6].

The uniqueness of this case lies in the rare coexistence of several severe complications - recurrent endoleaks, progressive renal dysfunction, and irreversible urological damage - occurring together in a relatively young patient with stage 4 CKD.

Acute kidney injury following endovascular aneurysm repair (EVAR)

AKI is a well-known complication of EVAR, with an incidence of up to 19%, especially in patients with reduced baseline eGFR or hypercholesterolemia.

Importantly, this renal insult was independently associated with significantly increased long-term mortality and cardiovascular morbidity during follow-up.

Pathophysiology includes contrast nephropathy, renal microembolization, hemodynamic instability, and direct injury to renal arteries. Ischemia-reperfusion, inflammatory response, and comorbidities such as CKD further increase the risk. [6]. In this patient, several of these risk factors coexisted, including stage 4 CKD, multiple contrast exposures, renal artery stent obstruction, and hemorrhagic complications, all of which contributed to recurrent AKI episodes and dialysis requirements.

The cumulative impact of both hemodynamic and obstructive mechanisms, combined with procedural burden, likely led to a cascade of renal insults. This highlights the importance of proactive renal protection strategies, particularly in vulnerable patients with complex aortoiliac pathology. Earlier use of contrast-sparing imaging, such as contrast-enhanced ultrasound (CEUS) or non-contrast MRI, might have reduced renal injury. More aggressive preventive strategies, including preprocedural hydration and limiting contrast volume, could also have been beneficial.

Endoleak

Endoleak is one of the most common long-term complications following EVAR. It refers to the continued pressurization of the aneurysm sac despite the placement of an aortic stent graft, resulting from persistent blood flow entering the sac from various sources [7]. In 1998, White et al. classified endoleaks based on their origin and mechanism into five distinct types [8]. Our patient experienced two clinically significant forms: type Ib, followed later by type Ia.

Type I involves direct blood flow into the aneurysm sac due to incomplete sealing between the stent graft and the vessel wall. Type Ia arises proximally near the renal arteries, often due to short or angulated necks or progressive dilatation. Type Ib occurs distally at the iliac arteries, usually from inadequate graft extension or iliac degeneration [8].

Notably, late type I endoleaks are typically detected between 34 and 52 months post-EVAR, often during routine surveillance imaging. Their clinical relevance lies in the fact that they are associated with a significantly increased risk of aneurysm rupture (4-7.5% within 2 years) and a high rate of late conversion to open surgical repair.

Type II occurs due to retrograde blood flow into the aneurysm sac via collateral vessels, most commonly lumbar arteries or the IMA. It is the most prevalent type of endoleak, constituting over 50% of all reported cases [9].

In this patient, these two endoleaks likely sustained high intraluminal pressure within the aneurysm sac, which could impair adjacent vascular structures, including renal and ureteral branches. Additionally, progressive aneurysmal dilatation and graft compression may have led to direct mechanical obstruction of the right ureter.

In a similar case, bilateral ureteral obstruction has been described. This confirms that EVAR-related complications may extend beyond vascular issues [10]. Interventions to treat endoleaks also carry urological risks, as illustrated by reported iatrogenic ureteral injuries during embolization [11].

Management required multiple complex procedures: relining with additional stent grafts, embolization, surgical revision, and aneurysmoraphy. Each intervention carried its own risks, including hemorrhage, infection, contrast exposure, and renal compromise.

Long-term imaging surveillance after EVAR

After EVAR, lifelong imaging follow-up is recommended. Patients with complex anatomy, early endoleak, or an enlarging aneurysm sac are at the highest risk. According to the Society for Vascular Surgery, computed tomography angiography (CTA) should be performed at 1, 6, and 12 months, and then yearly if stable [12]. In selected patients, CEUS or non-contrast MRI are useful alternatives that limit radiation and nephrotoxicity [13, 14]. Several studies suggest CEUS has sensitivity comparable to CTA for detecting type I and II endoleaks. It is especially useful in patients who cannot receive iodinated contrast [15].

Our patient demonstrates that endoleaks may appear many years after EVAR, requiring further interventions. This observation is consistent with the long-term data reported in 2024 and underscores the importance of lifelong surveillance and regular reassessment of treatment strategies [16].

EVAR vs open surgery

In young patients (<70), the selection of treatment requires careful consideration of long-term outcomes. EVAR is associated with lower early postoperative mortality (within the first 6 months); nevertheless, this benefit diminishes over time. After 8 years, both total and aneurysm-related mortality were significantly higher in the EVAR group compared to open repair [17].

In younger patients, life expectancy exceeds the time window during which EVAR offers a survival benefit. Furthermore, the endovascular approach necessitates lifelong imaging surveillance and carries a notable risk of late complications, particularly secondary aneurysm sac rupture.

Open repair, while more invasive, offers long-term durability, fewer reinterventions, and has demonstrated superior late survival outcomes in multiple trials [17-19].

Our patient met anatomic criteria for EVAR and was likely considered an ideal candidate; his relatively young age raises the question of whether open surgical repair would have been a more appropriate option.

This case underscores the importance of evaluating every patient for both EVAR and open surgical repair, with individualized consideration of anatomical features, life expectancy, and comorbidities [2]. In retrospect, open repair or use of a full-length aorto-bi-iliac stent graft might have reduced the risk of recurrent endoleaks and multiple reinterventions.

## Conclusions

This case underscores the complexity of managing abdominal aortic and iliac aneurysms in patients with severe risk factors. Despite the minimally invasive nature of EVAR, the risk of serious long-term complications - particularly endoleaks and renal deterioration - remains significant. Type IA and IB endoleaks contributed to progressive aneurysmal dilatation, ureteral compression, and recurrent episodes of AKI, necessitating multiple surgical and endovascular reinterventions. These complications highlight the critical importance of vigilant postoperative surveillance, proactive endoleak management, renal function protection, and coordinated multidisciplinary care in high-risk AAA patients.

From a broader perspective, this case also underscores the value of maintaining open surgical repair capabilities within vascular training programs. As the reliance on endovascular techniques grows, a decline in exposure to open procedures may compromise the ability to offer truly individualized treatment in complex or anatomically unsuitable cases.

Importantly, this case illustrates that treatment selection should always consider patient age, life expectancy, and anatomical factors - EVAR is not universally the better option.
